# Application of statistical methodology for the optimization of l-glutaminase enzyme production from *Streptomyces pseudogriseolus* ZHG20 under solid-state fermentation

**DOI:** 10.1186/s43141-023-00618-2

**Published:** 2023-11-24

**Authors:** Zuhour Hussein Wardah, Hiral G. Chaudhari, Vimalkumar Prajapati, Gopalkumar G. Raol

**Affiliations:** 1 Department of Microbiology, Shri Alpesh N. Patel PG Institute of Science and Research, Sardar Patel University, Vallabh Vidyanagar, Anand, Gujarat 388001 India; 2 Division of Microbial and Environmental Biotechnology, Aspee Shakilam Biotechnology Institute, Navsari Agricultural University, Athwa Farm, Ghod Dod Road, Surat, Gujarat 395007 India; 3 Shri R. P. Arts, Shri K.B. Commerce and Smt, BCJ Science College, Khambhat, 388620 Gujarat India

**Keywords:** l-glutaminase, *S*. *pseudogriseolus* ZHG20, Solid-state fermentation, Media optimization, RSM

## Abstract

**Background:**

Actinomycetes are excellent microbial sources for various chemical structures like enzymes, most of which are used in pharmaceutical and industrial products. Actinomycetes are preferred sources of enzymes due to their high ability to produce extracellular enzymes. l-glutaminase has proven its essential role as a pharmaceutical agent in cancer therapy and an economic agent in the food industry. The current study aimed to screen the potent l-glutaminase producer and optimize the production media for maximum enzyme yield using one factor at a time (OFAT) approach and statistical approaches under solid-state fermentation (SSF).

**Results:**

Out of 20 actinomycetes strains isolated from rhizosphere soil, 5 isolates produced extracellular l-glutaminase. One isolate was chosen as the most potent strain, and identified as *Streptomyces pseudogriseolus* ZHG20 based on 16S rRNA. The production and optimization process were carried out under SSF, after optimization using OFAT method, the enzyme production increased up to 884.61 U/gds. Further, statistical strategy, response surface methodology (RSM), and central composite design (CCD) were employed for the level optimization of significant media component (*p* < 0.05), i.e., wheat bran, sesame oil cake, and corn steep liquor which are leading to increase 3.21-fold l-glutaminase production as compared to unoptimized media.

**Conclusions:**

The presented investigation reveals the optimization of various physicochemical parameters using OFAT and RSM-CCD. Statistical approaches proved to be an effective method for increasing the yield of extracellular l-glutaminase from *S*. *pseudogriseolus* ZHG20 where l-glutaminase activity increased up to 1297.87 U/gds which is 3.21-fold higher than the unoptimized medium using a mixture of two solid substrates (wheat bran and sesame oil cake) incubated at pH 7.0 for 6 days at 33 °C.

## Background

Nowadays, there is an accelerated need to discover a novel antitumor drug and enhancement of a new therapeutic compound with high specificity and low side effects [1]. Recently, microbial enzymes were discovered to play a significant role in cancer treatment by depriving the cancer cells of essential nutrients [2]. Therapeutic enzymes derived from microorganisms (Actinomycetes, fungi and bacteria) are used to treat cancer and other severe disorders due to their substrate binding with high affinity as well as specificity [3].

l-glutaminase enzyme from microbial sources is approved as therapeutic enzyme which is effective against acute lymphocytic leukemia and various malignancies [4]. The great importance of microbial l-glutaminases as a pharmaceutical and food industrial agent is because they are simple to manufacture, stable and affordable, and they are conceivably used in cancer therapy [5]. l-glutaminase from *Streptomyces canarius* has showed noticeable efficacy against various human cancer cells. The study reported that the enzyme has a significant efficiency against human epithelial cells (HeLa cells), and human liver cancer cell line (Hep-G2), while the growth of breast cancer cell line (MCF-7 cells) was not affected, and slight cytotoxic impact is present on human colon carcinoma cell line (HCT-116), and macrophage cell line (RAW 264.7 cells) [6]. l-glutaminase also has antiviral activity against human immunodeficiency virus (HIV); it reduces the activity of serum reverse transcriptase of HIV. The inhibition of replication of HIV in infected cells was observed by l-glutaminase produced by *Pseudomonas* sp. 7A [7].

In the food industry, l-glutaminase has proven its role in flavor enhancement in fermented food by increasing l-glutamic acid content [8]. Additionally, it is used to minimize the generation of acrylamide in fried meals which occurs due to high temperature [9]. Actinomycetes produce a variety of compounds with different structures which are used as agrochemicals, pharmaceuticals, and industrial products such as enzymes [10]. Production of l-glutaminase from actinomycetes has been reported by Muthuvelayudham & Viruthagiri [11] and Aly et al. [12].

Various nutritional and physicochemical factors are affecting the l-glutaminase production, and the optimization of those factors leads to enhanced enzyme yield and make it more stable for specific application [6]. Additionally, actinomycetes were also reported as one of the better microbial sources for l-glutaminase, and several studies have been done on l-glutaminase production from *Streptomyces* sp. [13, 14]. The optimization of enzyme production from potent isolates to obtain maximum yield with better properties for a specific application owing to anticancer activity and the industrial agent is thus under research contentiously [15, 16]. In the present investigation, we aimed to screen l-glutaminase producing actinomycetes isolated from rhizosphere soil, as well as examine the appropriate fermentation process for enzyme production and optimize the culture conditions using one factor at a time (OFAT) and statistical approach to avail maximum enzyme yield.

## Methods

### Actinomycetes isolation and screening of l-glutaminase production

The actinomycetes strains were isolated from rhizosphere soil collected from a banana farm located in Anand (22°34′02.9′′N 72°53′52.4′′E), Gujarat, India. Actinomycetes are a major part of the rhizosphere’s microbial communities, which are crucial for recycling organic matter and breaking down complicated polymer mixtures in dead animals, plants, and fungi to produce a variety of extracellular enzymes for the environment. About 1 g of soil was suspended in 100 mL sterilized distilled water and then serially diluted up to 10^6^ dilutions. 0.1 mL of aliquot of dilution 10^3^ to 10^6^ were spread on Actinomycetes isolation agar (AIA). The isolation was done according to Wang et al. [17] with minor changes. The components of AIA include: (g/L) 2.0 C_20_H_27_FN_2_; 0.1 l-Asparagine; 4.0 C_3_H_5_NaO_2_; 0.5 K_2_HPO_4_; 0.1 MgSO_4_; 0.001 FeSO_4_, and agar 15 g. The plates were then incubated for 7 days at 30 ± 2 °C. The plate assay method (PAM) was used for primary screening. Actinomycetes were transferred on minimal glutamine agar (MGA) medium with addition of phenol red (0.012 g/L) as indicator. The plates were kept at 28 °C for 5 days. They were observed for the development of pink zone around the bacterial growth [18].

The secondary screening of l-glutaminase producing actinomycetes was done under submerged fermentation (SmF) by culturing them in 100 mL of minimal glutamine broth (MGB). All flasks were incubated at 28 ℃ for 5 days at 150 rpm. After centrifugation at 10,000 rpm for 15 min at 4 ℃, the supernatant was collected and used as an enzyme source for enzyme assay. l-glutaminase quantification was carried out by measuring the release of ammonia from hydrolyzing the amino acid l-glutamine using Nesslerization method. Ammonium standard graph was plotted using ammonium sulfate stock solution. Then, 0.1 mM to 1.0 mM of stock solution was taken in 10 different tubes serially and they were made up to 4.5 mL with phosphate buffer with pH 7.0. The blank was made up of 1 mL of double distilled water and 4.5 mL with phosphate buffer. Further, 400 µL of Nessler’s reagent was added, and the absorbance of the solution was determined at 450 nm and then the standard curve was drawn. The standard graph was used to calculate the concentration of ammonia released throughout the study. One unit of enzyme activity was defined as the amount of enzyme required to release 1 µmol of ammonia per minute per mL. l-glutaminase enzyme was assayed as described by More et al. [16]. After the enzyme assay, spectrophotometrically the optical density was measured at 450 nm. The isolate giving the highest l-glutaminase activity was selected for enzyme production.

### Identification of actinomycetes strain

The morphological characterization of isolate AC6 and AC20 was performed based on their phenotypic characteristics including colony size, color, shape, elevation, and microscopic characterization by examining the gram’s stain reaction followed by molecular identification based on 16S rRNA sequences. And the phylogenetic tree was concluded by comparing the sequence with the existing sequence data available through the gene bank database of the National Center for Biotechnology Information (NCBI) by MEGA-X software (11.0 version).

### l-glutaminase production and extraction

Two fermentation processes were used for l-glutaminase production, solid-state, and submerged fermentation. The inoculum was prepared by inoculating 150 mL nutrient broth with tested actinomycetes culture and incubated in incubator shaker for 5 days at 28 ℃ and 150 rpm. For l-glutaminase production under submerged fermentation (SmF), 2 mL of inoculum were added to 100 mL of sterile minimal glutamine broth (MGB) medium. The flask was incubated at 28 ℃ for 5 days at 150 rpm followed by l-glutaminase extraction at 10,000 rpm for 15 min at 4 ℃ using cooling centrifuge.

l-glutaminase production under solid-state fermentation (SSF) was carried out in a 250 mL Erlenmeyer flask containing 5 g of wheat bran (WB) as substrate and moistened with a moistening solution. The components of moistening solution include (g%) 0.5 l-glutamine; 0.5 Na_2_HPO_4_; 0.2 K_2_HPO_4_; 0.01 MgSO_4_; 0.1 NaCl; and corn steep liquor (CSL) as nitrogen source at concentration of 0.2 mL % (v/v). pH was adjusted to 6.5 using 0.1 N NaOH and HCL. The substrate and moistening solution were autoclaved separately at 15 lbs at 121 ℃ for 15 min, and then the moistening solution was added to the flask at the time of inoculation. Inoculum was 6 pieces of well-grown actinomycetes from the agar plates of selected strain; the pieces were made by sterilized cork borer (9.5 mm). After incubation for 5 days at 28 ℃, l-glutaminase was extracted using 20 mL of 0.1 M phosphate buffer (pH 7.0) and filtered using a sterile muslin cloth. The filtered extract was subjected to centrifugation and the clear supernatant was used as a crude enzyme for determining l-glutaminase activity and total protein. The protein content in the crude enzyme was determined by performing Lowry’s method by Lowry et al. [19]. Bovine serum albumin BSA was used as a standard.

### Optimization of SSF parameters for enhancing l-glutaminase production by Streptomyces pseudogriseolus ZHG20

#### OFAT optimization

It is also called one-variable-at-a-time, a technique used for testing factors, by changing one factor at a time rather than using multiple factors simultaneously. All experiments for l-glutaminase production optimization were performed under SSF. Various process parameters such as agro-residues, used as substrate and their particle size, l-glutamine concentration, nitrogen source, moisture ratio, pH, temperature, and incubation time were optimized using the one factor at a time approach (OFAT).

#### Media optimization

Different media with different compositions and concentrations for the same microorganism support the production of various by-products because of various metabolism pathways. The productivity of l-glutaminase is influenced by the type of factors used and their concentrations. In our study, different agro-industrial wastes were used as solid substrates to support microbial growth and l-glutaminase production, i.e., wheat bran (WB), sugarcane bagasse (SCB), rice bran (RB), sesame oil cake (SOC), Bengal gram husk (BGH), and pigeon pea seed husk (PPSH). Five grams of each substrate (coarse and fine particles) were added to 250 mL Erlenmeyer flask separately, moistened with a moistening solution containing: (g%) (w/v) 0.01% MgSO_4_.7H_2_O, 0.2% KH_2_PO_4_, 0.09% NaCl, 0.5 Na_2_HPO_4_, and CSL as nitrogen source at a concentration of 0.2 mL % (v/v); the flasks and moistening solution were separately sterilized at 15 lbs at 121 ℃ for 15 min. The moistening solution and substrate were combined at the time of inoculation with bacterial culture; all the flasks were incubated at 28 ℃ for 5 days. The effect of the addition of SOC along with WB in different concentrations from 1 to 5 g was studied. The effect of particle size of substrate by grinding it to 2000 > Particle Size (PS) > 1000, 1000 > PS > 500, 500 > PS > 250, 250 > PS > 150, and 150 > PS > 0.053 (µm) using mortar pestle, and the size of substrate particles was determined using different sizes of sieve ranging from 1000 to 0.053 µm. In separate experiments, the substrate with different particle size were used to examine the effect of each particle size on l-glutaminase production.

The impact of moisture ratio on production of l-glutaminase from 1: 2 to 1: 6 (w/v) was examined. l-glutamine acts as a nitrogen source and induces the synthesis of l-glutaminase. As the l-glutaminase production is influenced by increasing or decreasing l-glutamine concentration in production media, the effect of different concentrations of l-glutamine from 0.1 to 0.5 g% (w/v) was studied. Various nitrogen source, i.e., ammonium sulfate, urea, potassium nitrate, yeast extract, and corn steep liquor, were evaluated to determine the best nitrogen source and its concentration to obtain higher yield of l-glutaminase enzyme.

### Physical parameters optimization

To determine the optimum temperature for l-glutaminase, the production media was inoculated in 5 flasks (250 mL Erlenmeyer flask) containing 8 g of WB, and SOC (5:3) (w/w) moistened with 20 mL of moistening solution having 0.2% CSL (v/v) as constant variables, autoclaved, and incubated in the different temperature range 27 °C, 30 °C, 33 °C, 36 °C, and 40 °C. For optimization of pH, five flasks containing production media with pH range from 5.0 to 9.0 were inoculated with bacterial culture and incubated at 33 °C for 5 days; pH was adjusted using NaOH and 1 N HCL using pH meter (μ pH system 360, Systronic, India). The inoculated production media was incubated for 8 days, and enzyme activity was checked every 24 h.

### Statistical optimization using RSM

After determination of the factors influencing l-glutaminase production by OFAT method, the three important factors vis., WB, SOC and CSL, were selected for further optimization using response surface methodology (RSM) and central composite design (CCD) to determine the optimum concentration that maximizes the enzyme production from *S*. *pseudogriseolus* ZHG20. RSM is practically ineffective for a wide range of parameters due to the large number of experiments required, but few factors (up to five) can be utilized that give optimum results [20, 21] as it is more feasible to carry out its validation.

The level of the significant parameters as shown from OFAT experiments and their influence on l-glutaminase production were analyzed and optimized by CCD two-level factorial face centered. The level of the 3 major parameters WB, SOC, and CSL were optimized, while the other factors, i.e., temperature, pH, incubation time, and moisture level, were kept constant according to the previous optimization process. In CCD design, the total number of combinations is 2^ k^ + 2 k + no, where k detects the number of factors and each factor has two levels (low and high), while (no) is the number of replications of the experiments at the central point. In CCD design, each factor was studied at 5 different levels (− α, − 1, 0, + 1, + α), axial points encoded as – α and + α, factorial points encoded as − 1 and + 1 and central point (0). Table 1 denotes the ranges of variables (low and high) estimated based on our previous experiments.

**Table 1:** Levels and ranges of independent variables used for RSM-CCD

Independent variable	Coded Level					
		− α	− **1**	0	+ 1	** + α**
Wheat bran (g%)	A	− 1.64	2.5	3.75	5	5.85
Sesame oil seed cake (g%)	B	− 0.31	1	2	3	3.68
Corn steep liquor (mL)	C	− 0.06	0.2	0.4	0.6	0.73

The full experiment design with their experimental level/values is displayed in Table 2. l-glutaminase activity (U/gds) was determined in 60 separate experimental runs; all experiments were done under SSF. Equation 1 is a second-order polynomial equation employed to analyze l-glutaminase production and the multiple regression procedure was used to fit the data into the equation.

**Table 2:** Central composite design table

Run	Wheat bran	Sesame oil seed cake	Corn steep liquor	Predicted	l-glutaminase activity U/gds
1	3.75	2	0.4	753.21	877.02
2	2.5	1	0.2	508.26	329.51
3	2.5	3	0.2	775.95	879.73
4	3.75	2	0.4	753.21	856.16
5	5	3	0.2	1211.69	1299.36
6	3.75	3.68	0.4	779.14	819.37
7	5	3	0.2	1211.69	998.01
8	3.75	2	0.4	753.21	881.81
9	2.5	1	0.2	508.26	564.73
10	5.85	2	0.4	968.55	1247.98
11	2.5	1	0.6	317.97	471.8
12	2.5	3	0.2	775.95	862.58
13	1.64	2	0.4	366.55	319.22
14	2.5	3	0.6	159.22	79.88
15	2.5	3	0.6	159.22	332.83
16	5	3	0.6	762.2	845.41
17	3.75	2	0.06	884.78	931.95
18	5.85	2	0.4	968.55	838.9
19	3.75	2	0.4	753.21	292.89
20	5	1	0.2	621.17	639.051
21	3.75	2	0.4	753.21	856.94
22	2.5	1	0.6	317.97	629.06
23	3.75	2	0.4	753.21	855.22
24	5	1	0.2	621.17	435.29
25	5	3	0.6	762.2	945.47
26	3.75	2	0.4	753.21	733.91
27	3.75	3.68	0.4	779.14	636.21
28	3.75	2	0.4	753.21	868.68
29	3.75	2	0.4	753.21	880.53
30	3.75	2	0.4	753.21	998.03
31	3.75	0.31	0.4	416.08	162.17
32	2.5	3	0.2	775.95	704.78
33	5	1	0.6	598.14	765.82
34	5	3	0.2	1211.69	1245.62
35	3.75	2	0.4	753.21	857.87
36	3.75	2	0.4	753.21	856.41
37	3.75	2	0.73	346.8	78.14
38	3.75	3.68	0.4	779.14	687.67
39	3.75	0.31	0.4	416.08	734.56
40	3.75	2	0.4	753.21	695.48
41	3.75	2	0.4	753.21	741.4
42	3.75	2	0.4	753.21	794.26
43	3.75	2	0.73	346.8	261.43
44	5	1	0.2	621.17	638.89
45	5.85	2	0.4	968.55	945.11
46	3.75	2	0.4	753.21	745.98
47	2.5	3	0.6	159.22	543.6
48	5	3	0.6	762.2	582.72
49	3.75	2	0.4	753.21	606.61
50	3.75	2	0.06	884.78	825.92
51	5	1	0.6	598.14	551.78
52	3.75	2	0.4	753.21	238.31
53	3.75	0.31	0.4	416.08	81.32
54	5	1	0.6	598.14	685.95
55	2.5	1	0.6	317.97	273.51
56	1.64	2	0.4	366.55	105.95
57	2.5	1	0.2	508.26	871.91
58	3.75	2	0.73	346.8	148.47
59	1.64	2	0.4	366.55	83.76
60	3.75	2	0.063	884.78	984.49

1 $$\mathrm{Yi }=\upbeta 0 + \sum \mathrm{ \beta iXi }+ \sum \mathrm{ \beta iiXi}2 + \sum \mathrm{ \beta ijXiXj}$$

Where Y = the predicted response, XiXj = independent variables (Effect the response Y), β0 = constant, Βi = the linear coefficient, Βii = the quadratic coefficient and Βij = interaction coefficient.

### Software and data analysis

RSM modeling and experimental design, result analysis, and interpretation were carried out using Minitab software version 18.0 (Minitab GmbH, Munich, Germany).

## Results

### Isolation of actinomycetes and assessment of l-glutaminase production

**Fig. 1:**
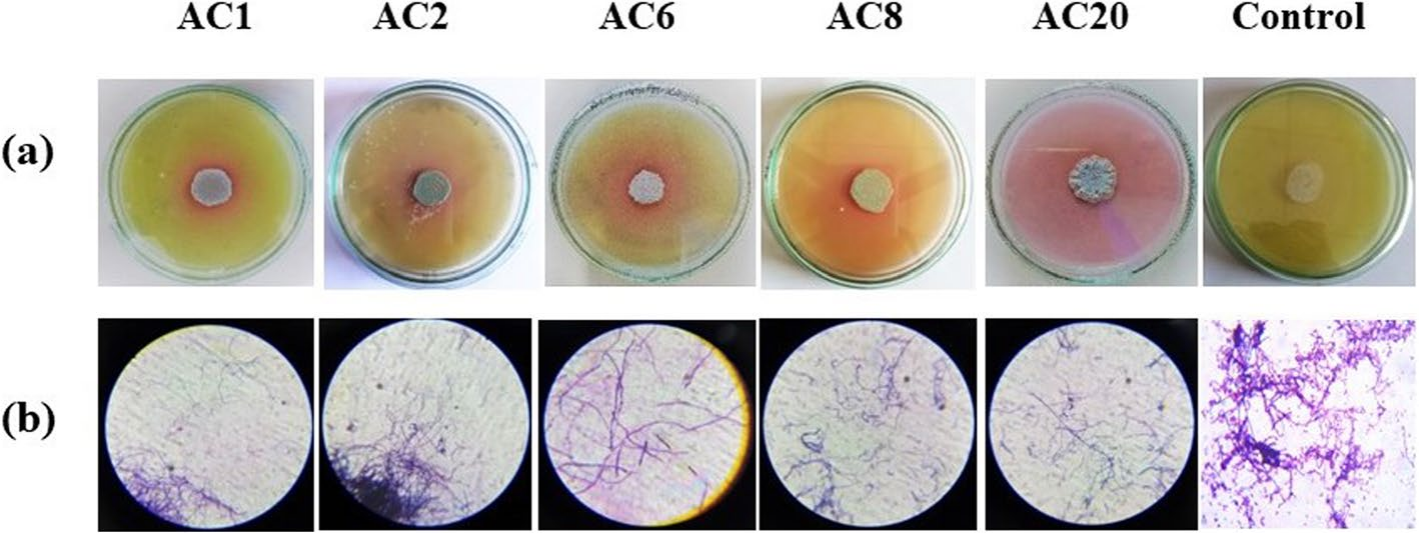
Primary screening of Actinomycetes isolates for extracellular l-glutaminase production. **a** Actinomycetes isolate with control. **b** Microscope photograph of tested isolates

**Fig. 2:**
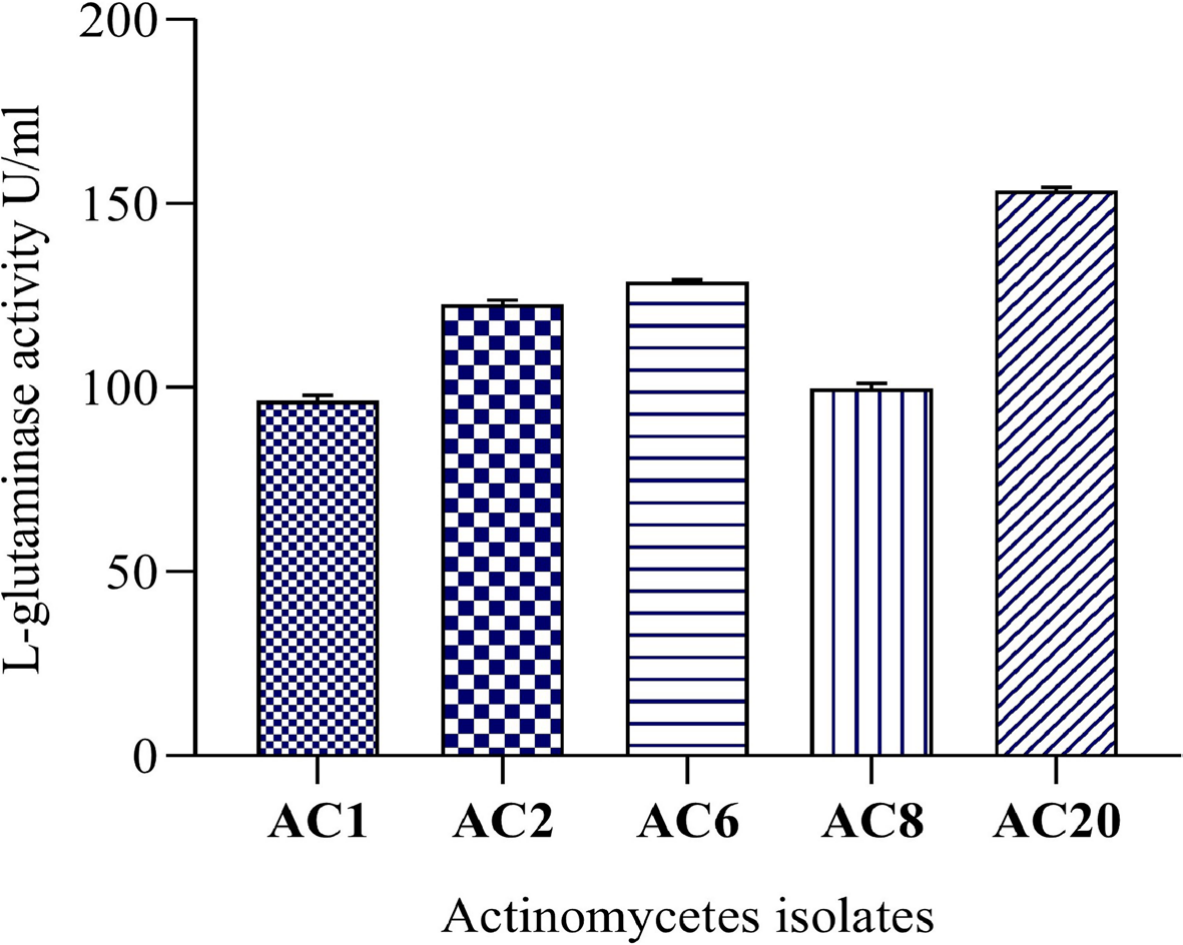
Secondary screening for l-glutaminase enzyme from selected actinomycetes isolates, AC20 strain was found to be the highest l-glutaminase producer

In the present study, the primary screening was done on twenty actinomycetes strains isolated from rhizosphere soil, out of these, 5 isolates produced l-glutaminase as shown on MGA medium (Fig. 1). The pink color zone indicates the release of ammonia due to the hydrolysis of amino acid l-glutamine by l-glutaminase and this indicates a positive result. All the 5 strains have different morphological characteristics and were selected for secondary screening by quantitative estimation of l-glutaminase under SmF. Two strains showed maximum l-glutaminase activity, AC6 and AC20. Using Nessler’s method, a quantitative screening for enzyme production was conducted. The maximum enzyme production was found by the same two actinomycetes strains, and a harmonious relationship was observed between the qualitative and quantitative screening. Although the strains AC6, AC20 have little difference in their values, AC20 showed significant result (maximum enzyme activity and good growth on media) with highest enzyme activity (153.06 U/mL) (Fig. 2) and was employed for future studies.

### Identification of actinomycetes strain

**Fig. 3:**
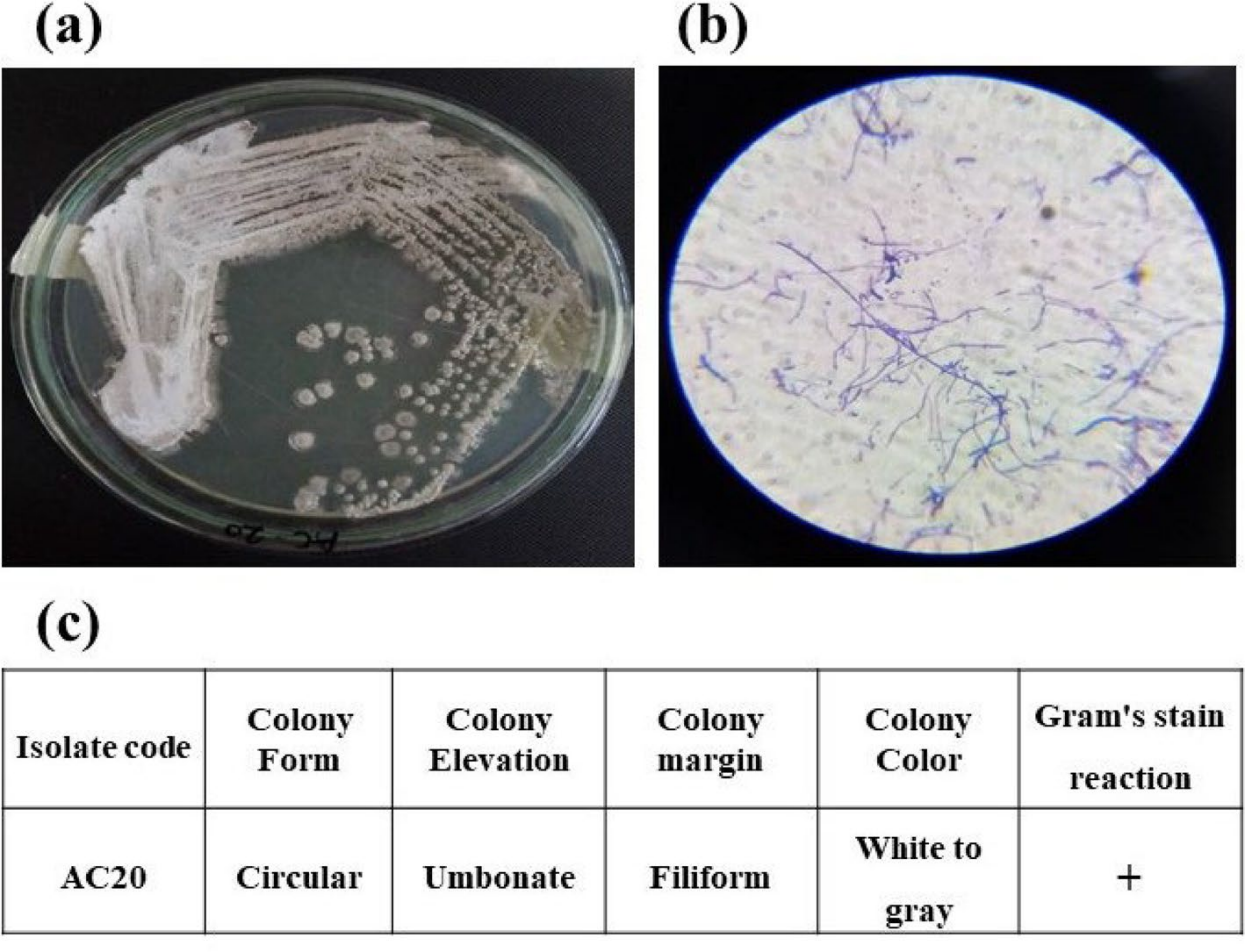
Morphological characterization and microscopic examination of potent isolate AC20. **a** AC20 on AIA, **b** microscope photograph, and **c** morphological characteristics

**Fig. 4:**
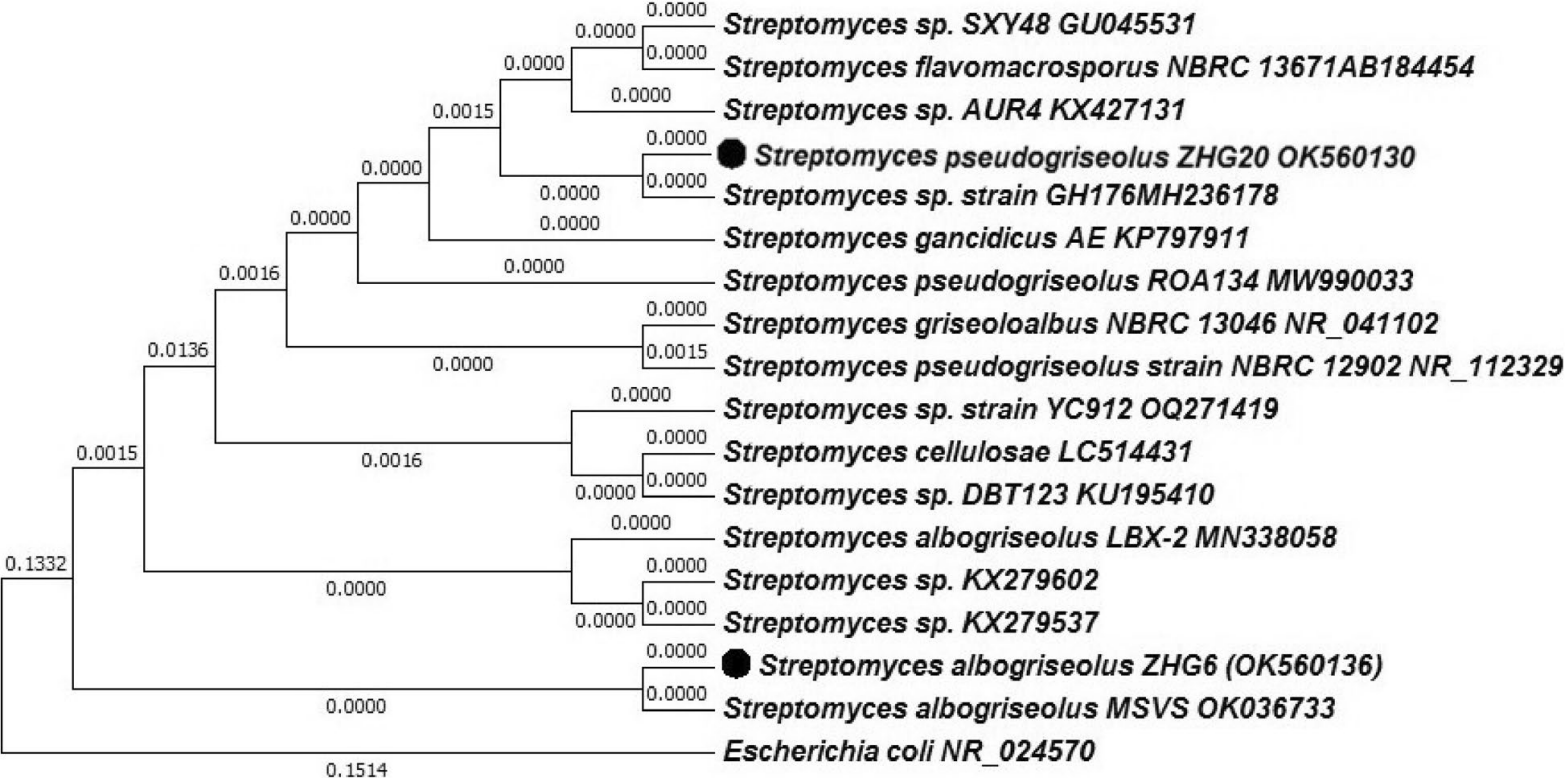
Phylogenetic tree based on 16S rRNA sequences of *S. albogriseolus* ZHG6 and *S*. *pseudogriseolus* ZHG20 and other related strains

The morphological and microscopic characterization of isolate AC20 was performed based on its phenotypic characteristics including colony size, color, shape, elevation, and gram stain reaction. On AIA plate, AC20 colonies were small in size, gray to white in color, powdery form, gram-positive, and filamentous under a microscope (Fig. 3). Based on 16S rRNA sequences, the sequences of AC6 and AC20 were compared with the similar sequences in GenBank. The results revealed that the isolates AC6 and AC20 were similar to *Streptomyces albogriseolus* with 98.70% and *Streptomyces pseudogriseolus* with 99.85% similarly respectively. The sequences were then deposited in GenBank as *S*. *albogriseolus* ZHG6 and *S*. *pseudogriseolus* ZHG20 with an accession number OK560136 and OK560130 respectively. The phylogenetic tree was inferred by using the neighbor-joining system with 1000 bootstrap by MEGA-X software (11.0 version) (Fig. 4); the sequences of AC6 and AC20 are presented in Table 3.

**Table 3:** 16S rRNA sequences of *S*. *pseudogriseolus* ZHG20 and *S*. *albogriseolus* ZHG6

Actinomycets isolate	Accession number	16S rRNA sequence
*Streptomyces pseudogriseolus* ZHG20	**(OK560130)**	CGCTGGCGGCGTGCTTAACACATGCAAGTCGAACGATGAACCACTTCGGTGGGGATTAGTGGCGAACGGGTGAGTAACACGTGGGCAATCTGCCCTGCACTCTGGGACAAGCCCTGGAAACGGGGTCTAATACCGGATACTGATCATCTTGGGCATCCTTGGTGATCGAAAGCTCCGGCGGTGCAGGATGAGCCCGCGGCCTATCAGCTTGTTGGTGAGGTAACGGCTCACCAAGGCGACGACGGGTAGCCGGCCTGAGAGGGCGACCGGCCACACTGGGACTGAGACACGGCCCAGACTCCTACGGGAGGCAGCAGTGGGGAATATTGCACAATGGGCGAAAGCCTGATGCAGCGACGCCGCGTGAGGGATGACGGCCTTCGGGTTGTAAACCTCTTTCAGCAGGGAAGAAGCGAAAGTGACGGTACCTGCAGAAGAAGCGCCGGCTAACTACGTGCCAGCAGCCGCGGTAATACGTAGGGCGCGAGCGTTGTCCGGAATTATTGGGCGTAAAGAGCTCGTAGGCGGCTTGTCGCGTCGGTTGTGAAAGCCCGGGGCTTAACCCCGGGTCTGCAGTCGATACGGGCAGGCTAGAGTTCGGTAGGGGAGATCGGAATTCCTGGTGTAGCGGTGAAATGCGCAGATATCAGGAGGAACACCGGTGGCGAAGGCGGATCT
*Streptomyces albogriseolus* ZHG6	**(OK560136)**	ATAGTTTGACCTGGTCAGGCGACGCTGGCGGCGTGTTAACACAGCAATCGAACGAGAACATTCGGTGGGGATTAGTGGCGAACGGGTGAGTAACACGTGGGCAATCTGCCCTGCACTCTGGGACAAGCCCTGGAAACGGGGTCTAATACCGGATACTGACCCGCTTGGGCATCCAAGCGGTTCGAAAGCTCCGGCGGTGCAGGATGAGCCCGCGGCCTATCAGCTTGTTGGTGAGGTAATGGCTCACCAAGGCGACGACGGGTAGCCGGCCTGAGAGGGCGACCGGCCACACTGGGACTGAGACACGGCCCAGACTCCTACGGGAGGCAGCAGTGGGGAATATTGCACAATGGGCGAAAGCCTGATGCAGCGACGCCGCGTGAGGGATGACGGCCTTCGGGTTGTAACCTCTTTCGCAGGGAAGAACGAAAGTGACGGTACCTGCAGAAGAAGCGCCGGCTAACTACGTGCCAGCAGCCGCGGTAATACGTAGGGCGCGAGCGTTGTCCGGAATTATTGGGCGTAAAGAGCTCGTAGGCGGCTTGTCACGTCGGTTGTGAAAGCCCGGGGCTTAACCCCGGGTCTGCAGTCGATACGGGCAGGCTAGAGTTCGGTAGGGGAGATCGGATTCCTGGTGTAGCGGTGAAATGCGCAGATATCAGGAGGAACACCGGTGGCGAAGGCGGATCTCTGGGCCGATACTGACGCTGAGGACGAAAGCGTGGGGAGCGAACAGGATTAGATACCCTGGTAGTCCACGCCGTAAACGGTGGGCACTAGGTGTGGGCGACATTCCACGTCGTCCGTGCCGCACTAACGCATTAAGTGCCCCGCCTGGGGAGTACGGCCGCAAGGCTAAACTC

### l-glutaminase production and its optimization

**Fig. 5:**
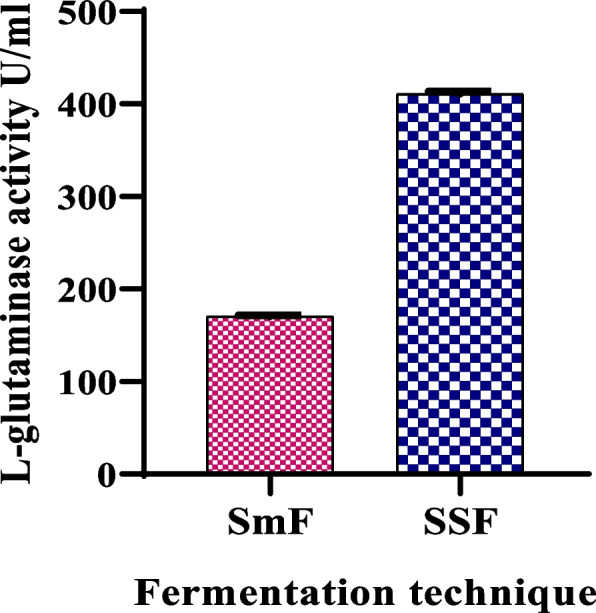
l-glutaminase production by *S. pseudogriseolus* under SmF and SSF

Production of l-glutaminase was done under SmF and SSF; both fermentation processes have different fermentation conditions for bacterial growth and enzyme production. In SSF conducted using WB as substrate, the enzyme activity was 411.12 U/gds which is 2.40-fold higher than the enzyme activity produced under SmF (Fig. 5). Therefore, for further production and optimization, SSF was selected.

### Optimization of l-glutaminase production media

#### Media optimization

**Fig. 6:**
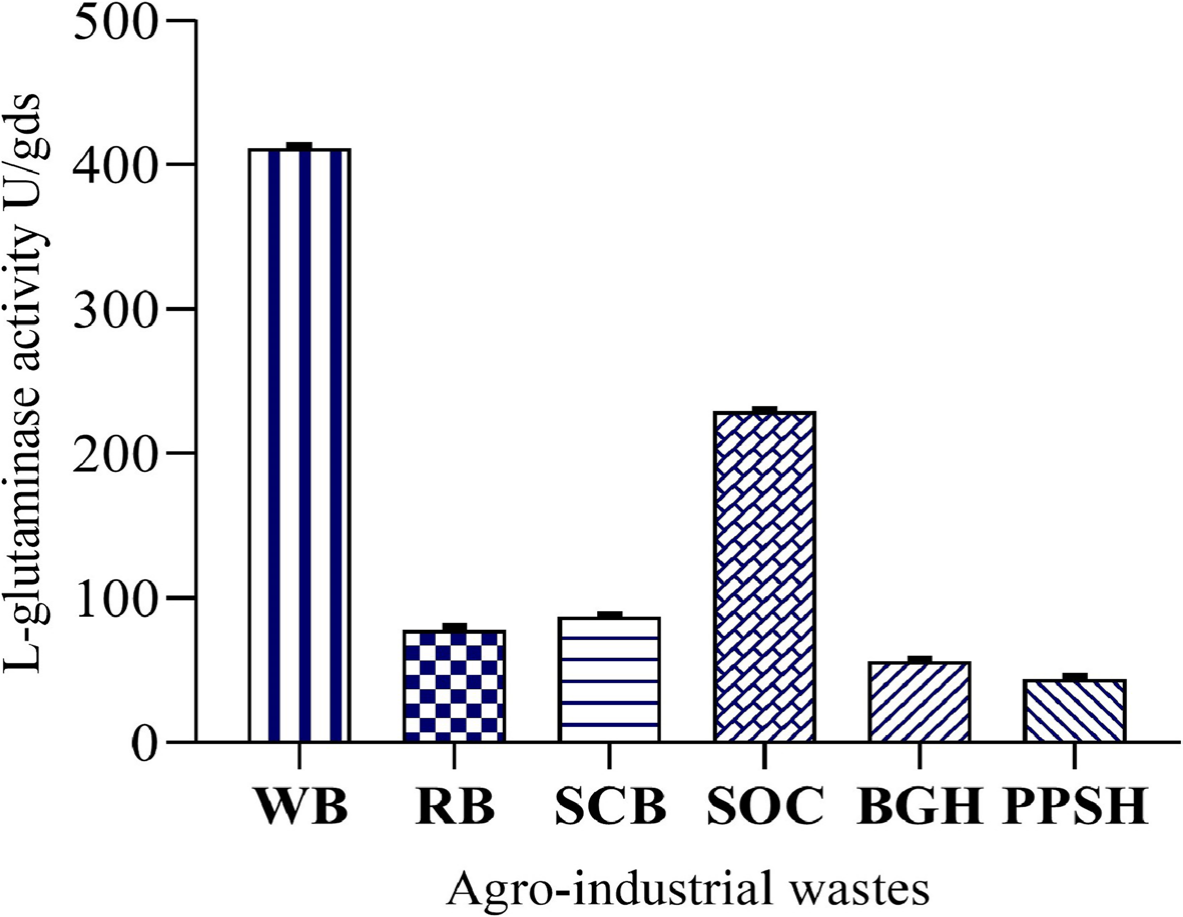
Effect of different substrates on lglutaminase production, wheat bran (WB), rice bran (RB), sugarcane bagasse (SCB), sesame oil cake (SOC), Bengal gram husk (BGH), and pigeon pea seed husk (PPSH)

In the present study, WB showed maximum l-glutaminase activity followed by SOC, while the lowest enzyme activity was observed with PPSH (Fig. 6). The addition of SOC along with WB as a carbon source at a concentration of (3:5) (WB: SOC) (w/w) led to increase in enzyme productivity from 411.12 U/mL to 551.82 U/gds compared to WB alone. The particle size of any substrate used in SSF has an eminent effect on microbial growth and enzyme production [20]. Hence, the substrates used in SSF were grinding to 2000 > Particle Size (PS) > 1000, 1000 > PS > 500, 500 > PS > 250, 250 > PS > 150, and 150 > PS > 0.053 (µm) and used in separate experiments to examine the effect of each particle size on l-glutaminase productivity. We found that the particle size of substrate significantly affected l-glutaminase production. The 1000 > PS > 500 (µm) particle size of substrate led to enhanced l-glutaminase production (589.74 U/gds) (Table 4). The l-glutamine concentration for l-glutaminase production was determined to be 0.3% (g/v); it increased the enzyme yield to 644.51 U/gds (Table 5). Among different nitrogen sources tested, CSL at a concentration of 0.2 mL% (v/v) led to the highest enzyme activity (657.01 U/gds) (Tables 5 and 6) compared to other concentrations. The moisture level in SSF is a very important factor that influenced the success of the fermentation process as well as enzyme production. l-glutaminase production was examined by modifying the initial moisture level of production media from 1:2 to 1:6 using a moistening solution, and it was observed that 1:5 moisture level led to the highest l-glutaminase activity (665.57 U/gds).

**Table 4:** Effect of particle size of substrate on l-glutaminase production by *S*. *pseudogriseolus* ZHG20

PS (μm)	EA (U/gds)	Specific activity U/mg
2000 > PS > 1000	551.64 ± 0.58	6.41 ± 1.23
1000 > PS > 500	589.21 ± 1.22	7.22 ± 1.08
500 > PS > 250	509.38 ± 1.14	6.16 ± 0.34
250 > PS > 150	374.33 ± 1.09	4.62 ± 1.76
150 > PS > 0.053	206.24 ± 0.93	2.55 ± 1.56

*PS* particle size, *EA* enzyme activity

**Table 5:** Effect of glutamine concentration on l-glutaminase production by *S. pseudogriseolus* ZHG20

Glu (g)	EA (U/gds)	Specific activity U/mg
0.1	374.63 ± 0.89	4.41 ± 0.34
0.2	401.40 ± 0.64	4.92 ± 0.89
0.3	644.20 ± 0.81	7.79 ± 1.34
0.4	433.83 ± 1.12	5.36 ± 1.09
0.5	391.51 ± 0.55	4.8 ± 1.13

*EA* enzyme activity (l-glutaminase), *Glu* glutamine

**Table 6:** Effect of different nitrogen sources on l-glutaminase production by *S. pseudogriseolus* ZHG20

NS (0.2 g)	EA (U/gds)	Specific activity U/mg
Ammonium sulfate	553.69 ± 1.94	6.50 ± 0.12
Potassium nitrate	403.96 ± 1.35	4.95 ± 1.23
Urea	390.52 ± 1.51	4.72 ± 1.03
Corn steep liquor	656.41 ± 1.25	7.73 ± 1.98
Yeast extract	595.87 ± 1.71	6.36 ± 1.02

*NS* nitrogen sources, *EA* enzyme activity (l-glutaminase)

### Physical parameters optimization

#### The effect of temperature

Temperature has an eminent effect on l-glutaminase production and its activity. At low temperature, the enzyme becomes active in form, whereas the enzyme will be deactivated at high temperature. Production media was incubated at different temperature range 27 °C, 30 °C, 33 °C, 36 °C, and 40 °C. The optimum temperature for enzyme production was found to be 33 °C, and the enzyme activity obtained was 627.6625 U/gds. Lowest enzyme activity was observed at 40 °C; the enzyme activity obtained was 101.1 U/gds.

#### The effect of pH

pH is one of the most important parameters which affect enzyme production as well as microbial growth. In the present study, the varying pH altered the enzyme production and the ideal pH for production of l-glutaminase by *S*. *pseudogriseolus* ZHG20 was found to be at pH 7.0 (652.93 U/gds). While increasing the pH value of the production media to alkaline conditions lead to reduction in the enzyme production to 6.451% (42.125 U/gds) at pH 9.0.

#### The effect of incubation time

l-glutaminase production was increased gradually with increasing the fermentation time over a period of up to 168 h (6 days) and the maximum yield was observed to be 884.61 U/gds. An additional incubation time beyond the optimum fermentation time led to the decrement in the l-glutaminase yield and bacterial growth. Table 8 depicts the effect of different factors on the L-glutaminase production by S. pseudogriseolus ZHG20.

### Result of RSM optimization

CCD was performed to determine the effect of the optimum level of significant factors and their interactions on l-glutaminase production by *S*. *pseudogriseolus*. The three variables WB, SOC, and CSL were selected for level optimization based on the outcomes of OFAT. The results obtained by CCD were analyzed by ANOVA. The predicted and observed response are presented in Table 2. It shows l-glutaminase production (U/gds) which corresponds to the combined effect of all three components within the given ranges. The regression equation produced using RSM for l-glutaminase is given below:

$$\mathrm{Y }= -\hspace{0.17em}58 + 93\mathrm{ A }+ 299\mathrm{ B }+ 611\mathrm{ C }-\hspace{0.17em}19.4\mathrm{ A}*\mathrm{A }-\hspace{0.17em}55.0\mathrm{ B}*\mathrm{B }-\hspace{0.17em}1215\mathrm{ C}*\mathrm{C}+ 64.6\mathrm{ A}*\mathrm{B }+ 167\mathrm{ A}*\mathrm{C }-\hspace{0.17em}533\mathrm{ B}*\mathrm{C}$$

where Y = response (yield of l-glutaminase (U/gds)), A = the concentration of WB (g), B = SOC (g), and C = CSL (mL %).

**Fig. 7:**
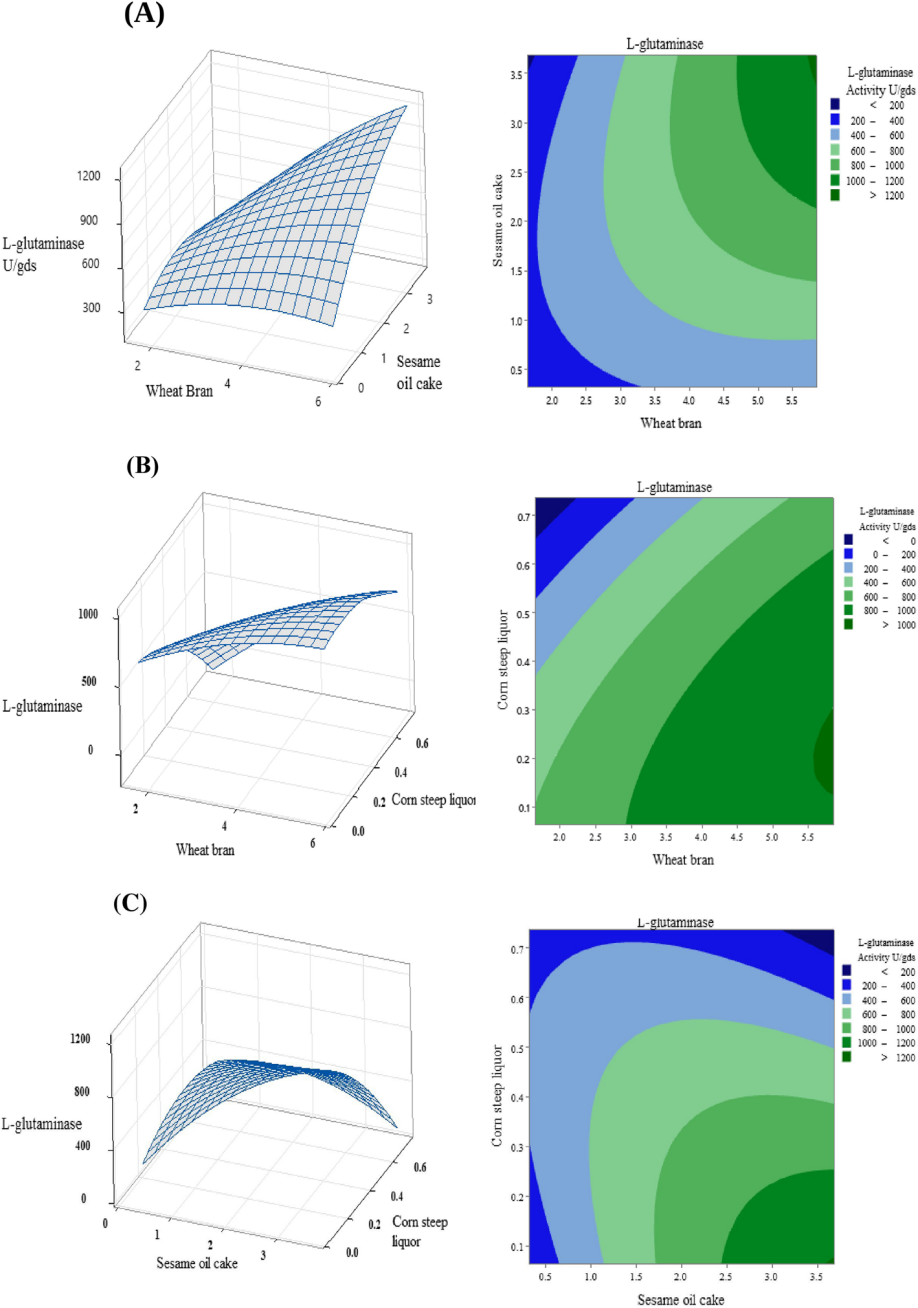
RSM graph (surface plots and contour plots) representing the influence of the variable on production of L-glutaminase, **A** Wheat bran and sesame oil cake, **B** wheat bran and corn steep liquor, **C** Sesame oil cake and corn steep liquor

The statistical significance was estimated by the analysis of variance (ANOVA) and *F* test for the quadratic model as presented in Table 9, which detects that the regression is significant for l-glutaminase production. In this study, *F* value of the quadratic model is 9.52 which means that the model is significant. The experimental *P* value is equal to or lower than 0.050, which represents that the model terms are significant. In our study, the terms A, B, C, AB, and BC are significant. The influence of independent variables on l-glutaminase production was studied by altering the concentration of two variables while keeping the other variable at constant. The 3D surface plots, and contour plots can be used to find the optimal levels of tested variables. In the present investigation, three response surface plots and three contour plots were obtained through RSM-CCD experiment and depicted in Fig. 7. These plots describe the interaction effect, main effect, and squared effect of the three independent variables used in the experimentation as well the behavior of l-glutaminase production by *S*. *pseudogriseolus* ZHG20 under solid-state fermentation. Figure 7A showing the effect of WB and SOC on l-glutaminase yield at different concentrations, the lower level of WB and SOC did not result in higher l-glutaminase yield while the lower level of WB and the higher level of SOC resulted in high l-glutaminase yield. The surface and contour plot shown in Fig. 7B describes the interaction between WB and CSL while keeping the SOC at constant level. The higher level of WB and the lower level of CSL resulted in high enzyme yield. The 3D response plot shown in Fig. 7C describes the interaction between SOC and CSL while keeping the WB at constant level; the lower level of CSL and higher level of SOC maximize the enzyme yield. The optimum level determined for each variable were 5 g wheat bran, 3 g sesame oil cake 0.2 mL corn steep liquor, and 0.3 g glutamine, at pH 7.0 and 33 ℃ for 6 days. Additional experiments were performed in triplicate using the above optimized medium; the l-glutaminase yield achieved was 1297. 87 U/gds with specific activity 12.32 U/mg.

## Discussion

In recent years, l-glutaminase production using microbial sources (Actinomycetes, bacteria, and fungi) has increased due to its effective role as an economical agent in the food industry, cancer therapy, and in biosensors as a monitoring agent to estimate l-glutamine level [22]. Many microorganisms have been reported as l-glutaminase producers and many reports are available on its production process [23, 24]. The isolation and screening purpose of l-glutaminase production from microorganisms is to find out the best enzyme producers which generates higher yield with unique properties to be used in medical and industrial applications (Tables 7, 8 and 9).

**Table 7:** Effect of corn steep liquor concentration on l-glutaminase production by *S. pseudogriseolus* ZHG20

CSL (mL)	EA (U/gds)	Specific activity U/mg
0.2	657.01 ± 0.99	7.71 ± 0.34
0.4	571.18 ± 0.71	6.07 ± 1.31
0.6	498.03 ± 0.75	6.02 ± 0.72
0.8	347.39 ± 1.01	4.29 ± 1.34
1	238.81 ± 0.88	2.95 ± 1.05

*CSL* corn steep liquor, *EA* enzyme activity (l-glutaminase)

**Table 8:** Effect of pH, temperature, and incubation time on l-glutaminase production by *S. pseudogriseolus* ZHG20

pH	EA (U/gds)	Specific activity U/mg	Temperature	EA (U/gds)	Specific activity U/mg	INC (h)	EA (U/gds)	Specific activity U/mg
5	130.46 ± 1.00	1.53 ± 1.07	27 ℃	463.51 ± 0.62	5.44 ± 1.01	24	17.89 ± 1.05	0.29 ± 1.03
5.5	92.20 ± 1.06	1.13 ± 0.56				48	25.12 ± 0.92	0.63 ± 1.55
6	193.34 ± 1.42	2.3 ± 0.34	30 ℃	616.32 ± 2.61	7.55 ± 1.67	72	51.60 ± 0.56	0.80 ± 0.34
6.5	588.46 ± 0.69	7.27 ± 1.78				96	66.81 ± 0.78	5.30 ± 0.14
7	652.78 ± 1.55	8.07 ± 1.09	33 ℃	627.45 ± 0.97	8.58 ± 0.84	120	428.78 ± 0.58	6.82 ± 0.74
7.5	640.58 ± 0.59	7.53 ± 1.23				144	551.71 ± 0.56	9.02 ± 2.01
8	551.44 ± 1.26	6.76 ± 1.22	36 ℃	560.26 ± 1.22	6.92 ± 0.28	168	884.96 ± 0.72	9.36 ± 1.79
8.5	33.68 ± 1.38	0.40 ± 0.79				192	857.02 ± 6.01	7.34 ± 1.54
9	42.76 ± 0.97	0.52 ± 0.16	40 ℃	101.34 ± 1.12	1.25 ± 1.81	216	821.78 ± 1.67	6.98 ± 2.78

*INC* incubation time

**Table 9:** Analysis of variance of quadratic model for l-glutaminase production by *Streptomyces pseudogriseolus* ZHG20

**Source**	**DF**	**SS**	**MS**	***F* value**	***P* value**	
Model	9	3,540,192	393,355	9.52	0.000	Significant
Linear	3	2,837,792	945,931	22.91	0.000	Significant
A	1	1,312,363	1,312,363	31.78	0.000	Significant
B	1	477,343	477,343	11.56	0.001	Significant
C	1	1,048,085	1,048,085	25.38	0.000	Significant
Square	3	231,333	77,111	1.87	0.147	
A^a^	1	39,660	39,660	0.96	0.332	
B^a^	1	130,849	130,849	3.17	0.081	
C^a^	1	102,058	102,058	2.47	0.122	
2-way interaction	3	471,067	157,022	3.8	0.016	Significant
AB	1	156,319	156,319	3.79	0.057	Significant
AC	1	41,960	41,960	1.02	0.318	
BC	1	272,788	272,788	6.61	0.013	Significant
Error	50	2,064,889	41,298			
Lack-of-Fit	5	450,079	90,016	2.51	0.441	Nonsignificant
Pure Error	45	1,614,810	35,885			
Total	59	5,605,081				

^a^*DF* degrees of freedom, *SS* sum of square, *MS* mean square, *A* wheat bran, *B* sesame oil cake, *C* corn steep liquor

In the present study, 20 strains of actinomycetes were isolated from rhizosphere soil and were checked for l-glutaminase production using the plate assay method (PAM) and MGA as a selective medium in the presence of phenol red as an indicator. Five isolates showed positive results as a zone of pink color formed around the colony. In the same line, bacterial strains, *Halomonas meridiana*, and *Bacillus* sp. DV2-37 were isolated from a particular environment and primary screening was done for l-glutaminase production by plate assay method [2, 25]. The strain which showed the highest l-glutaminase activity through secondary screening was selected and identified as *S*. *pseudogriseolus* ZHG20 based on 16S rRNA and was subjected for further evaluation. Similarly, actinomycetes strains such as *S*. *canarius* and *Streptomyces* sp. D214 were isolated and screened for l-glutaminase production and identified based on 16S rRNA [6, 12].

Actinomycetes are filamentous bacteria that can grow and penetrate the solid substrate and absorb the nutrition needed for their growth [26]; this property is much useful for l-glutaminase production under SSF. The WB was used as solid substrate and led to enzyme yield of 411.12 U/gds which was 2.40-fold higher than the enzyme yield under SmF. Kiruthika et al. reported the l-glutaminase production (236.67 U/mL) by *Bacillus subtilis* JK-79 strain using WB under SSF [27].

Different solid substrates are used for l-glutaminase production from various microorganisms [28]. The mixture of wheat bran and sesame oil cake supported the bacterial growth as well as enzyme productivity. Similarly, a mixture of Green Gram Husk (GGH) and SOC gave an enzyme activity (259.32 U/gds) by *Aspergillus wentii* MTCC 1901 [29]. Additionally, l-glutaminase production using WB and Bengal Gram Husk (BGH) by *Aspergillus flavus* MTCC 9972 under SSF was also reported by [28]. The temperature of the fermentation medium affects microbial growth as well as l-glutaminase synthesis and production [30]. The optimum temperature for l-glutaminase production by *S*. *pseudogriseolus* ZHG20 was reported to be at 33 ℃ (627.66 U/gds) but increasing the temperature led to decrease in enzyme production (101.1 U/gds). Mousumi and Dayanand reported the maximum l-glutaminase production from *Streptomyces enissocaesilis* DMQ-24 at 40 ℃ [14].

On the other hand, very alkaline or acidic pH can also inhibit the fermentation process, thus reducing the biosynthesis of l-glutaminase. In our study, we noticed that the optimum pH for enzyme production was found to be 7.0. Similar observations were documented for enzyme production under SmF by *Aspergillus oryzae* NRRL 32567 [31] and *Bacillus* sp. DV2-37 [25]. Incubation period is one of the most influential factors for any metabolite production, and the optimum incubation period for the fermentation medium is dependent on the type of bacterial strain and its abundance in the fermentation media. Maximum enzyme activity could be measured only after a certain incubation period, but prolonged incubation can also lead to depletion of the nutrients by microbes in the fermentation medium thereby resulting in inactivation of the secretory machinery of the enzymes. Maximum enzyme yield was obtained by *S*. *pseudogriseolus* ZHG20 after 6 days of incubation period. Balagurunathan et al. reported maximum l-glutaminase production by *Streptomyces olivochromogenes* was after 4 days [32]. Whereas Mousumi and Dayanand reported that the optimal incubation period for enzyme production was after 5 days by *S*. *enissocaesilis* DMQ-24 [14].

In the present investigation, we observed that, after optimizing the fermentation parameters for l-glutaminase production by *S*. *pseudogriseolus* ZHG20 using OFAT approach under SSF, the enzyme productivity was found to be 884.61 U/gds, i.e., 2.1-fold increment compared to unoptimized media. In the same line, compared to initial medium l-glutaminase production increased by 2.88-fold under SSF [27] and 3.48-fold by *Bacillus subtilis* JK-79 [33] under optimum conditions.

After determining the factors that influence l-glutaminase production by OFAT method, the three important factors vis., WB, SOC, and CSL, were selected for further optimization using RSM to determine the optimum concentration that maximizes the enzyme production from *S*. *pseudogriseolus* ZHG20. RSM-CCD helped in identifying the significant levels that influenced l-glutaminase production by *S*. *pseudogriseolus* ZHG20. SOC, WB, and CSL are the most factors affecting l-glutaminase production under solid-state fermentation. l-glutaminase production was reported by *Bacillus subtilis* JK-79; the highest enzyme activity (236.67 U/mL) was observed using WB under SSF [27]. While a combination of a solid substrate, *Aspergillus flavus* MTCC 9972 produced greater levels of l-glutaminase in a 59:41 ratio of Bengal gramme husk to wheat bran [28]. After optimization, the enzyme activity increased up to 1297.87 U/gds. RSM-CCD proved to be an efficient method to analyze and optimize the production of extracellular enzymes like l-glutaminase using microorganisms as enzyme source such as *Providencia sp* [34] and *Bacillus* sp. [35]. It has been also employed to identify the levels of significant variables that influencing the production of various enzymes such as Glucoamylase [20], β-galactosidase [21], l-Asparaginase [36], and Amylase [37].

## Conclusions

The current research revealed that the actinomycetes strain belonging to *Streptomyces* species, isolated from rhizosphere soil and identified as *S. pseudogriseolus* ZHG20 based on 16S rRNA, proved to be the potent bacterial isolate for l-glutaminase production. According to the thorough survey, it is the first report on l-glutaminase enzyme production using the actinomycetes strain *S*. *pseudogriseolus* ZHG20 and was yielding higher enzyme compared to other actinomycetes strains. l-glutaminase production was optimized using OFAT and statistical approach under SSF, both methods helped to identify the significant variables of production media and their optimum concentrations for maximum production of extracellular l-glutaminase. The maximum l-glutaminase yield was observed when the bacterial strain was allowed to grow on a mixture of two solid substrates (wheat bran and sesame oil cake) moistened with a moistening solution supplemented with 0.2% mL CSL (v/v), 0.3 g% glutamine, at pH 7.0 incubated at 33 ℃ for 6 days. After optimization, the enzyme activity was found to increase up to 1297.87 U/gds. The results revealed that the statistically optimized fermentation process could be an effective method for increasing extracellular l-glutaminase productivity by the potent isolate *S*. *pseudogriseolus* ZHG20.

## Data Availability

Data are available upon request from the author.

## Abbreviations

SSF: Solid-state fermentation
SmF: Submerged fermentation
OFAT: One factor at a time
HeLa cells: Human epithelial cells
Hep-G2: Human liver cancer cell line
MCF-7 cells: Breast cancer cell line
HCT-116: Human colon carcinoma cell line
RAW 264.7 cells: Macrophage cell line
HIV: Human immunodeficiency virus
AIA: Actinomycetes isolation agar
PAM: Plate assay method
MGA: Minimal glutamine agar
MGB: Minimal glutamine broth
CSL: Corn steep liquor
WB: Wheat bran
SCB: Sugarcane bagasse
RB: Rice bran
SOC: Sesame oil cake
BGH: Bengal gram husk
PPSH: Pigeon pea seed husk
RSM: Response surface methodology
CCD: Central composite design
GGH: Green gram husk
